# Low Molecular Mass Myocardial Hyaluronan in Human Hypertrophic Cardiomyopathy

**DOI:** 10.3390/cells8020097

**Published:** 2019-01-29

**Authors:** Christina E. Lorén, Christen P. Dahl, Lan Do, Vibeke M. Almaas, Odd R. Geiran, Stellan Mörner, Urban Hellman

**Affiliations:** 1 Cardiology, Heart Centre, Department of Public Health and Clinical Medicine, Umeå University, 901 85 Umeå, Sweden; christinae.loren@gmail.com (C.E.L.); lan.do@umu.se (L.D.); stellan.morner@umu.se (S.M.); 2 Department of Cardiology, Oslo University Hospital Rikshospitalet, 0424 Oslo, Norway; christen.peder.dahl@rr-research.no (C.P.D.); vibalm@ous-hf.no (V.M.A.); 3 Department of Clinical Medicine, UiT, the Arctic University of Norway, 9019 Tromsø, Norway; 4 Research Institute of Internal Medicine, Oslo University Hospital Rikshospitalet, 0372 Oslo, Norway; 5 Faculty of Medicine, University of Oslo, 0318 Oslo, Norway; ogeiran@medisin.uio.no; 6 Department of Thoracic and Cardiovascular Surgery, Oslo University Hospital Rikshospitalet, 0424 Oslo, Norway

**Keywords:** hypertrophic cardiomyopathy, hyaluronan, metabolomics, GEMMA, glucose

## Abstract

During the development of hypertrophic cardiomyopathy, the heart returns to fetal energy metabolism where cells utilize more glucose instead of fatty acids as a source of energy. Metabolism of glucose can increase synthesis of the extracellular glycosaminoglycan hyaluronan, which has been shown to be involved in the development of cardiac hypertrophy and fibrosis. The aim of this study was to investigate hyaluronan metabolism in cardiac tissue from patients with hypertrophic cardiomyopathy in relation to cardiac growth. NMR and qRT-PCR analysis of human cardiac tissue from hypertrophic cardiomyopathy patients and healthy control hearts showed dysregulated glucose and hyaluronan metabolism in the patients. Gas phase electrophoresis revealed a higher amount of low molecular mass hyaluronan and larger cardiomyocytes in cardiac tissue from patients with hypertrophic cardiomyopathy. Histochemistry showed high concentrations of hyaluronan around individual cardiomyocytes in hearts from hypertrophic cardiomyopathy patients. Experimentally, we could also observe accumulation of low molecular mass hyaluronan in cardiac hypertrophy in a rat model. In conclusion, the development of hypertrophic cardiomyopathy with increased glucose metabolism affected both hyaluronan molecular mass and amount. The process of regulating cardiomyocyte size seems to involve fragmentation of hyaluronan.

## 1. Introduction

Hypertrophic cardiomyopathy (HCM) is a disorder with a prevalence of 1/500 and an annual mortality of approximately 1% [1]. It is a monogenic inherited disease associated with cardiac dysfunction and life threatening arrhythmias [2,3]. HCM exhibits a wide phenotypic variability ranging from asymptomatic to severe symptoms and is an important cause of sudden cardiac death in young adults and athletes. At the cellular level, HCM is characterized by patches of cardiomyocyte hypertrophy, cardiomyocyte disarray, interstitial fibrosis, and small vessel disease. 

When subjected to hemodynamic or metabolic stress, the heart returns to fetal metabolism and the fetal gene program where the cells prefer usage of glucose over fatty acid as source of energy [4]. It has been proposed that dysfunctional regulation of the glucose metabolism and cardiac energy metabolism is a prominent feature of the maladapted failing heart and HCM hearts [5,6]. A rise of glucose level results in an increased influx trough of the hexosamine biosynthetic pathway (HBP) [7], resulting in elevated levels of uridine diphosphate *N*-acetyl-d-glucosamine (UDP-GlcNAc).

A significant consequence of higher levels of UDP-GlcNAc is the increased synthesis of the extracellular matrix (ECM) glycosaminoglycan hyaluronan (HA), which has not previously been explored in HCM. HA is a polydisperse unbranched polymer that greatly varies in molecular mass ranging from 5 to 10,000 kDa, which makes it challenging to analyze. It is present in the ECM of all vertebrates, and is highly expressed during development, wound healing, and regeneration. HA is synthesized at the cellular membrane by the linkage of the UDP-sugar precursors d-glucuronic acid (GlcUA) and *N*-acetyl-d-glucosamine (GlcNAc), a reaction catalyzed by the membrane bound enzyme hyaluronan synthases (HAS). The HA synthesis is strongly dependent on the cytoplasmic concentrations of UDP-GlcNAc and UDP-GlcUA, and it has been shown that increased levels of these UDP-sugar precursors enhance HA synthesis [8,9]. 

Several reports have shown that diverse sizes of HA exert a wide spectrum of functions. In health and tissue homeostasis, HA is present as high molecular mass (MM) HA and has structural and hydrating features as well as an anti-inflammatory effect [10]. On the contrary, low MM HA has been shown to have a pro-inflammatory effect [11]. In addition, many receptors and extracellular proteins have been shown to bind HA, creating a molecular network with a wide range of structural and signaling properties. Degradation of HA is mainly carried out by hyaluronidases (HYAL, CEMIP) or by reactive oxygen species (ROS). Recent work has revealed HA as a driving factor in the development of fibrosis by stimulating both fibroblast proliferation, differentiation, and motility [12]. Furthermore, it has been shown that high MM HA depolarizes the membrane in cell cultures in a concentration dependent manner, which could be reversed by cell surface digestion of HA by hyaluronidase [13]. If this is also true for cardiomyocytes, it could potentially change the cardiac action potential leading to rhythm disturbances and arrhythmias [14].

We have earlier shown elevated gene expression of *HAS 1*, *2* and the HA receptor *CD44* as well as increased cardiac levels of HA correlating with pro-hypertrophic gene expression, using a rat model for cardiac hypertrophy [15,16]. We have also identified a crosstalk between cultured cardiomyocytes and fibroblasts resulting in increased HA synthesis in the fibroblasts [17]. In addition, HA staining was stronger in human cardiac tissues from HCM patients compared to autopsy material from previously healthy individuals [18], and in the rat heart HA occurs around myofibrils [19].

In this study we further investigate HA in HCM. We observed an altered metabolism of HA in HCM and changes in molecular mass distribution of HA corresponding with cardiomyocyte size. 

## 2. Materials and Methods

### 2.1. Human Tissue Samples

Tissue aliquots from human septal myocardium were obtained during surgery with basal septal myectomy from five patients with hypertrophic obstructive cardiomyopathy. Two of the patients were diagnosed with coronary disease. None were diagnosed with diabetes or hypertension. Characteristics are presented in Table 1.

Control (non-failing) human left ventricular, right ventricular, and septal tissue was obtained from five sex- and age-matched subjects whose hearts were rejected as cardiac donors for surgical reasons. The cause of death of donors was cerebrovascular accident or carbon monoxide poisoning, and none had a history of heart disease or known previous medication. Myocardium from these subjects was kept on ice for 1 to 4 h before tissue sampling. All cardiac tissues were snap-frozen in liquid nitrogen and stored at −80 °C until use. 

### 2.2. Rat Model for Hypertrophy

Cardiac tissues were analyzed from a rat model of cardiac hypertrophy from a study published several years ago (2008) [15]. Due to the newly developed GEMMA (gas-phase electrophoretic molecular mobility analyzer) analysis of HA mass distribution, it was now possible to perform HA mass analysis on the small pieces of cardiac tissue saved from the previous study [20]. The surgical procedure of the rats has been described elsewhere [15]. Briefly, rats were anesthetized with 0.2 mL pentobarbital intra-abdominal and after abdominal incision, a titanium clip of 1.15 mm inner diameter was administered on the aorta proximal to the renal arteries on male Wistar rats (n = 3). Age-matched control rats (n = 3) were sham operated, i.e., subjected to the same procedure but without the administration of a clip on the aorta. After being anesthetized with 0.4 mL pentobarbital, the rats were sacrificed at 1 and 42 days after operation and their hearts were harvested. 

### 2.3. Compliance with Ethical Standards

All procedures performed in studies involving human participants were in accordance with the ethical standards of the Regional Health Authorities of South-Eastern Norway and with the 1964 Helsinki declaration and its later amendments. Informed consent was obtained from all individual participants included in the study. 

The animal study was performed in Paris at Inserm U689 and all procedures performed in studies involving animals were in accordance with animal welfare regulations, and the study protocol was approved by the Ethical Committee of Inserm. All investigations conformed to guidelines set by the French Ministry of Agriculture and the Guide for the Care and Use of Laboratory Animals published by the US National Institutes of Health.

### 2.4. ^1^H High-Resolution Magic Angle Spinning Nuclear Magnetic Resonance (HR MAS NMR) Spectroscopy

Tissue samples were thawed at room temperature and kept on ice during preparation. Each tissue sample (20–30 mg wet weight) was inserted into disposable 30-µL Teflon NMR inserts followed by the addition of deuterium oxide to the insert to complete the required volume and homogenize insert contents. Inserts were then packed into a 4 mm zirconia MAS rotor (40 μL capacity). All the NMR experiments on tissue samples were carried out on a 500 MHz MAS-NMR spectrometer (Bruker, Billerica, MA, USA) at 300 K. 

### 2.5. NMR Data Processing and Analysis

Spectra were imported into MATLAB (R2015a) (MathWorks Inc., Natick, MA, USA) integrated using in-house developed scripts and normalized by the sum of all intensities. All metabolites in human cardiac tissues were identified using Chenomx NMR Suite 7.7 software (Chenomx Inc., Edmonton, AB, Canada) with full resolution NMR data and a standard two-dimensional (2D) NMR experiment on a selected sample. For identifying metabolites contributing to the discrimination between groups, the normalized ^1^H-NMR data were uploaded to SIMCA (version 14, Umetrics, Umeå, Sweden) for orthogonal partial least squares-discriminant analysis (OPLS-DA). The spectral variables were scaled to unit variance, and 7-fold internal cross-validation was performed to evaluate the quality of the resulting statistical models by considering the diagnostic measures R2 and Q2. Potential metabolites were selected based on the variable importance in projection (VIP) score > 1.0.

### 2.6. Immunohistochemistry

Before paraffin embedding, human cardiac tissues were fixed in 4% buffered formaldehyde for 48 h in room temperature. Paraffin sections (4 µm) were then mounted on SuperFrost Plus Slides (Thermo Fisher Scientific Inc., Waltham, MA, USA) and dried overnight at 37 °C. Prior to staining, sections were deparaffinized in xylene, rehydrated in series of graded ethanol, and washed in PBS. To visualize HA only, deparaffinized histological sections were blocked for 30 min using 2% bovine serum in PBS. Samples were incubated with a biotinylated HA binding protein (HABP) probe overnight at 4 °C [21]. HABP was prepared at the Institute of Medical and Physiological Chemistry, University of Uppsala, at a concentration of 100 µg/mL and was diluted 1:40 [22]. The samples were rinsed three times in PBS and then incubated with streptavidin 488 (1:500, Thermo Fisher Scientific Inc., MA, USA) for 1 h at room temperature.

To visualize HA and collagen (I, III, and VI, respectively) in the same section, antigen retrieval was performed by boiling the sections in a citrate buffer in a microwave oven for 9 min followed by cooling for 30 min before washing in water for 5 min. The sections were then blocked for 30 min using 2% bovine serum in PBS. Finally, samples were incubated overnight at 4 °C with the primary reagents HABP and rabbit anti collagen I (ab292 1:200, Abcam, Cambridge, UK), rabbit anti collagen III (ab7778 1:400, Abcam, UK), or rabbit anti collagen VI (ab6588 1:400, Abcam, UK), respectively. After washing in PBS, the samples were incubated with streptavidin 488 (1:500, Thermo Fisher Scientific Inc., MA, USA) and donkey anti rabbit 594 (1:500, Thermo Fisher Scientific Inc., MA, USA). Nuclei were stained with Hoechst 33,343 (1:5000, Thermo Fisher Scientific Inc., MA, USA), and slides were mounted in ProLong Gold Antifade (Thermo Fisher Scientific Inc., MA, USA). Phalloidin-iFluor 594 1:1000 (ab176757, Abcam, UK) was used as a cell marker. All stainings were visualized using the Zeiss LSM 710 laser-scanning confocal microscope (Zeiss, Oberkochen, Germany).

### 2.7. Cardiomyocyte Area Analysis

Cardiomyocytes were visualized by iFluor 594-labeled phalloidin (1:1000, Abcam, UK) staining actin and ECM was visualized by staining collagen III (ab 7778 1:400, Abcam, UK). Each slide was objected to 10 images. Morphometric analysis was performed using the software Fiji (http://fiji.sc). An average area value from each heart was calculated by the use of the measurements of 30–50 cells containing a central nucleus. Statistical analysis was performed using a two-tailed Student t test assuming unequal variances. Each pixel was calibrated to 0.83 μm according to Zeiss confocal data. Fluorescence images were collected at 20× objective using Zeiss LSM 710 laser-scanning confocal microscope (Zeiss, Oberkochen, Germany).

### 2.8. HA Molecular Mass Distribution

Cardiac tissue samples, wet weight 27–112 mg, were dried (n = 5 in each group) and homogenized. Proteins and nucleic acids were digested with proteinase K (Sigma-Aldrich, St. Louis, MO, USA), benzonase nuclease (Sigma-Aldrich, MO, USA), and chondroitinase ABC (Sigma-Aldrich, MO, USA) on three consecutive days. At the end of each day, chloroform was added to each sample and the extracted aqueous phase was dialyzed against 0.1 M NaCl using Amicon Ultra 3K concentration units (Millipore, Burlington, MA, USA) followed by overnight precipitation in 99% ethanol (EtOH). Samples were then loaded on anion exchange mini spin columns (Thermo Fisher Scientific, MA, USA) and centrifuged to wash out sulphated glycosaminoglycans and remaining non-HA contaminants, based on NaCl-binding. Finally, to remove salt the sample was dialyzed against 20 mM ammonium acetate (pH 8.0) in Amicon Ultra 3K concentration units. HA molecular mass analyses were performed using a nano-electrospray gas-phase electrophoretic molecular mobility analyzer (GEMMA) (TSI Corp., Shoreview, MN, USA). 

Each sample of purified HA (n = 5 in each group) was scanned three times in the GEMMA and the final size distribution spectrum was a sum of the three scans. The raw counts from the GEMMA spectrum were calibrated according to the previously described method [23]. The molecule diameter analyzed in the GEMMA was converted to molecular mass by analyzing HA standards ranging from 30 to 2500 kDa (Hyalose LLC, OC, USA). The relation between area under the curve (AUC) in the GEMMA spectrum to the HA concentration enables an estimation of the relative concentration of different MM of HA. Counts on the Y-axis correspond to the number of detected molecules and the X-axis to the MM of HA. Number of counts were normalized to the dry weight of the sample. Due to the physical properties of HA and the shape dependence of the GEMMA method, the analysis achieves a good separation of low MM HA up to ca. 100 kDa whereas the resolution for higher MM is poorer. The relative amount of HA with an MM less than about 100 kDa cannot be compared with HA of an MM greater than 100 kDa [23].

In this study, we have defined low MM HA as a mass up to 50 kDa. HA extracted from cardiac tissue was degraded with hyaluronidase from *Streptomyces hyalurolyticus* (Sigma-Aldrich, MO, USA) and reanalyzed to test for extraction specificity.

### 2.9. RNA Extraction and qRT-PCR

To obtain RNA, the cardiac tissues were homogenized in Qiazol lysis agent and with beads using Precellys lysing kit (Bertin Instruments, Montigny-le-Bretonneux, France) and purified using the RNeasy plus Universal Mini Kit (QIAGEN, Waltham, MA, USA). Reverse transcription was performed with 1 µg of total RNA using the High Capacity RNA to DNA kit (Thermo Fisher Scientific, MA, USA). The extracted RNA and cDNA concentration, respectively, were quantified using a NanoDrop Spectrometer ND-1000 (NanoDrop, Thermo Fisher Scientific Inc., Waltham, MA, USA).

The real-time quantitative PCR was performed on a 7900 HT Fast Real-Time PCR system (Thermo Fisher Scientific, MA, USA) using 1 µg cDNA, TaqMan® Gene Expression Assays, and 1 µL Gene Assay Mix for the genes *HAS1-3*, *HYAL 1* and *2*, *CEMIP*, *CD44*, *VCAN*, and *TSG6* (Thermo Fisher Scientific, MA, USA). *GAPDH* (Thermo Fisher Scientific, MA, USA) was used as an endogenous reference gene. Forty cycles of amplification were performed. The gene of interest was normalized to the reference gene using the ΔCt method [24]. 

### 2.10. Statistical Analysis

For metabolomics statistics, non-parametric Mann–Whitney U test with Benjamini–Hochberg correction using in-house software written and compiled in MATLAB (MathWorks Inc., Natick, MA, USA) was used. For analysis of gene expression and relative amount of water, low and high MM HA non-parametric independent Mann–Whitney U test was performed using the SPSS statistic software (version 25, IBM, Armonk, NY, USA). *P* values of less than 0.05 were considered to be significant. Factor analysis was performed with the principal components method to analyze the correlation matrix and two factors were extracted.

Generation of box plots for cardiomyocyte area was performed using SPSS statistic software.

## 3. Results

### 3.1. Metabolomic Analysis of Cardiac Tissue from HCM Patients and Non-Failing Hearts

It has been shown that increase in the synthesis of HA is strongly dependent on the concentration of UDP-GlcNAc and UDP-GlcUA. A higher level of these UDP hexosamines leads to increased synthesis of ECM HA. Therefore, we wanted to analyze the metabolomics and known metabolites in this process in HCM and healthy patients.

NMR was performed using cardiac tissues from non-failing septum and left ventricle (n = 10) and basal septal myectomies from HCM patients (n = 5). UDP hexosamines were identified as a merged multiple peak with an almost 2-fold increase. UDP-GlcUA can be formed from *myo*-Inositol, which was increased 1.5-fold in HCM. Glucose, on the other hand, was decreased 1.5-fold. Lactate was increased about 3.5-fold in HCM. Glutamate and glutamine showed a 3- and 2-fold increase, respectively, in HCM, and glutathione levels were increased almost 2-fold in HCM. Finally, fatty acid levels were decreased 1.5–2-fold in HCM, as shown in Table 2. These results support the proposal that dysfunctional regulation of the glucose metabolism may be involved in the development of HCM. An interesting observation is the increase in glutamate and glutamine, which are substrates for gluthation known to have a protective function in the regulation of ROS.

### 3.2. Assessment of HA Morphology in Basal Septal Myectomies and Non-Failing Septum

To better understand the nature of development of HCM, we wanted to investigate the morphology and distribution of HA and collagen in HCM and non-failing myocardial tissues. Therefore, HA and collagen distribution was compared in cardiac tissue from HCM patients and non-failing controls subjected to immunohistochemistry and studied in the confocal microscope.

**Figure 1:**
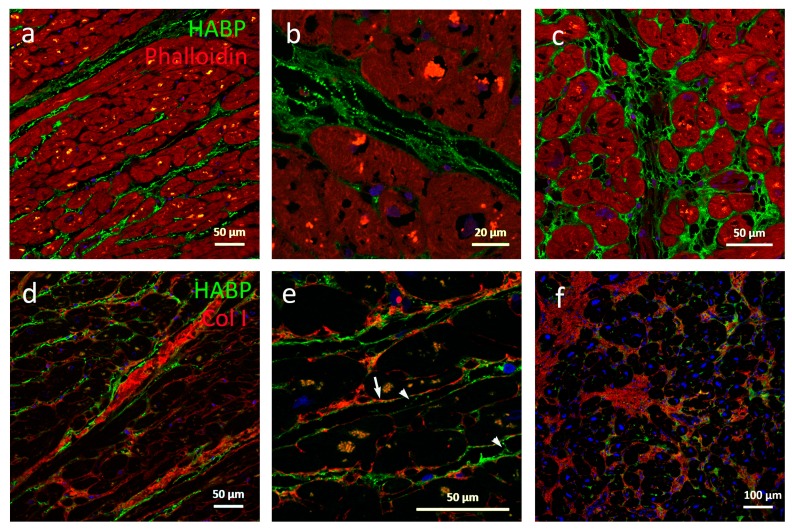
Morphological distribution of HA in cardiac tissue. Cardiomyocytes from non-failing septum (healthy controls) (**a**,**b**,**d**–**e**) n = 5 and HCM (**c**,**f**) n = 5. (**a**) In non-failing hearts, HA form long fine fibers in the perimysium running parallel to myofibers. (**b**) Sometimes these fibers are aligned with stained dots of HA forming an appearance of a string of pearls. (**d**) Collagen coil structures are surrounded by, but exclude, HA. (**e**) HA is located immediately adjacent to collagen weaves (arrow), and form strand-like structures between fibers (arrowheads). (**c**) In HCM, individual cardiomyocytes are separated by HA, which is also more abundant regularly filling the perimysium forming large patches with enlarged ring-like structures. (**f**) In areas with expanded ECM with fibrosis, there were patches where collagen and HA were mutually excluded. Hyaluronan binding protein (HABP, green), cardiomyocytes (phalloidin, red), nuclei (Hoechst, blue) (**a**–**c**). Hyaluronan binding protein (HABP, green), collagen I (Col I, red), nuclei (Hoechst, blue) (**d**–**f**). Large pale red dots inside cells are artefacts due to auto fluorescence.

In non-failing hearts, the natural intermyofibrillar space is aligned with HA forming long fine fibers running parallel to the myofibers, as shown in Figure 1a. These fibers were connected with HA staining strands occasionally seen to run adjacent to collagen strands, as shown in Figure 1e. Both HA fibers and strands were regularly covered with dots of HA, forming an appearance of a string of pearls, as shown in Figure 1b. HA fills the ECM around capillaries and larger vessels. Collagen I could be visualized around individual cardiomyocytes and in the weaves surrounding the myofibrils, as shown in Figure 1e. In contrast, HA staining was weak or absent between individual cardiomyocytes, but located immediately adjacent outside collagen weaves as well as covering collagen higher order structures such as coils, as shown in Figure 1d. 

Individual cardiomyocytes were separated or absent in the HCM tissue in areas with largely expanded and unorganized ECM, as shown in Figure 1f. In addition, these areas were filled with patches of HA. Also, the intermyofibrillar HA staining fibers and strands were thicker than in non-failing tissues, and in some samples, large, non HA staining holes were identified in areas with HA patches, as shown in Figure 1c. In some regions HA and collagen I were localized in the same patches, whereas in some areas within the same sample, regions were observed where HA and collagen I seemed excluded from each other, as shown in Figure 1f. Stained dots of HA were also less frequent in HCM myectomies. No gross difference in staining of collagen I, III, and VI was observed. These data suggest that HA levels, structure, and distribution play a role in the development of HCM. Since HCM is described as a “patchy” disease, the differential expression pattern of collagen related to HA may reflect different stages of progression in HCM.

### 3.3. Comparison of Cardiomyocyte Size 

**Figure 2:**
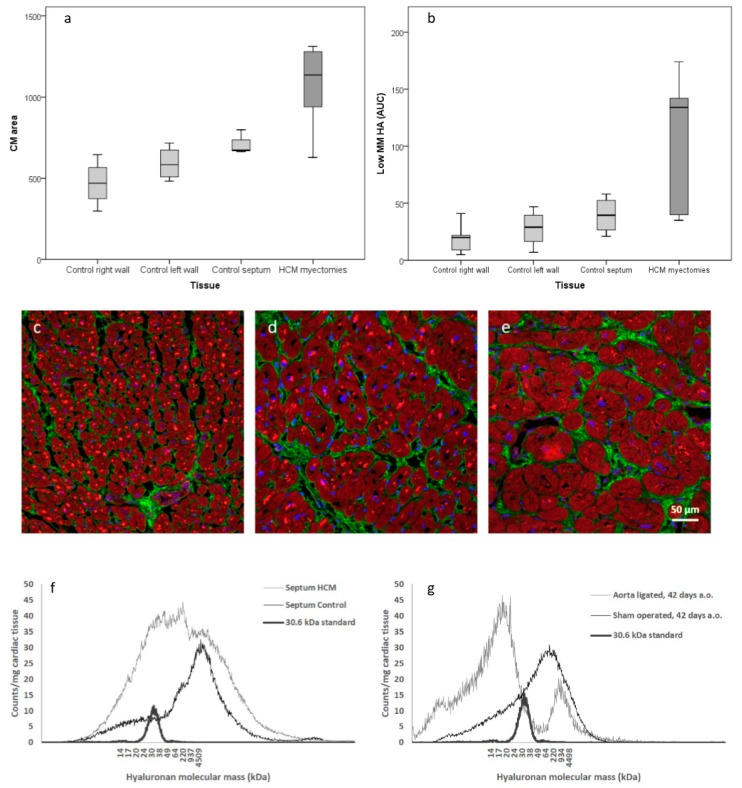
Cardiomyocyte (CM) size and amount of low molecular mass (MM) HA. Box plot illustrating cardiomyocyte size comparison between HCM and non-failing right wall septum and left wall, respectively (**a**). Box plot illustrating amount (corresponds to area under curve, AUC) of low MM HA in HCM and non-failing right wall septum and left wall, respectively (**b**). Immunohistochemistry of human heart tissues from right- (**c**), and left- (**d**) wall and HCM myectomies (**e**). The cardiomyocytes were larger in left ventricle than right wall. (**a**,**c**,**d**) The largest cells could be found in HCM myectomies which also contained the lowest MM HA (**b**). Analysis of HA molecular mass distribution, where HA was extracted from human (n = 5/group) and rat hearts (n = 3/group) and separated by gas-phase electrophoretic molecular mobility analyzer (GEMMA) according to molecular charge (**f**,**g**). In the myectomies, there was a significant increase of low MM HA (*P* < 0.001) (**f**). There was a clear shift from high MM HA to low MM HA in aorta ligated rats compared to sham operated rats 42 days after surgery (**g**). Cardiomyocytes (phalloidin, red), ECM (collagen III, green), nuclei (Hoechst, blue). Size bar in (**e**) is representative for all immunohistochemistry (**c**–**e**).

Since it is known that HCM is characterized by patches of cardiomyocyte hypertrophy, we wanted to further analyze the distribution of hypertrophic cardiomyocytes in the septum, right and left ventricle wall, respectively. Cardiomyocyte size from HCM patients and non-failing controls were histologically examined with confocal microscopy. Cell size determination showed that cardiomyocytes in right wall, left wall, and septum in controls were of significantly (*P* < 0.001) different sizes, as shown in Figure 2a. In addition, the cells of the right wall were less densely packed. No gross difference in morphology or distribution of HA was identified between the right wall and left wall, as shown in Figure 2c,d. In HCM, very large cardiomyocytes were identified which were significantly larger than cardiomyocytes in healthy left ventricles, as shown in Figure 2e,d, Table 3. 

### 3.4. Analysis of HA Mass Distribution

It has been reported that high MM HA has an anti-inflammatory effect in tissue homeostasis. In contrast, low MM HA has been reported to have a pro-inflammatory effect. Therefore, HA molecular mass in HCM and healthy hearts was investigated. Cardiac tissues from human subjects and from a rat model of induced hypertrophy [15] were subjected to GEMMA to determine HA mass distribution, as shown in Figure 2f,g, Table 3. 

In HCM myectomies there was an increased amount of low MM HA compared to left septum from non-failing controls, as shown in Figure 2b,f, Table 3. A difference could also be seen within controls, as shown in Figure 2b. Cardiac tissue with larger cardiomyocytes seems to contain more low MM HA and amounts of low MM HA correlated with the size of cardiomyocytes (*P* < 0.001, Pearson correlation coefficient = 0.792), as shown in Figure 2a,b. Surgically-induced cardiac hypertrophy revealed a clear shift from high MM HA to low MM HA in the aorta-ligated rats 42 days after surgery, as shown in Figure 2g. Samples degraded with hyaluronidase showed no remaining contaminations.

### 3.5. Gene Expression

To further understand the dynamics of HA metabolism, mRNA from HCM and non-failing human cardiac tissue was analyzed for the expression of *HAS1-3*, *HYAL 1-2*, *CEMIP*, *CD44*, *VCAN,* and *TSG6*. There was a 1.8-fold decrease of *HYAL2* (*P* = 0.003) and a 3.1-fold decrease of *HAS2* in myectomies compared to non-failing septum (*P* = 0.016). *HAS3* showed a 2.1-fold increase in myectomies (*P* = 0.008), as shown in Table 3. The other genes tested were all expressed although no significant statistical difference could be identified (data not shown). No significant difference between left and right ventricle gene expression was observed (data not shown). Levels of *HYAL2* and *HAS3* correlated to amounts of low MM HA in non-failing left ventricles (*P* = 0.009, Pearson correlation coefficient = 0.840 and *P* = 0.012, Pearson correlation coefficient = 0.822, respectively) but not in HCM myectomies.

**Figure 3:**
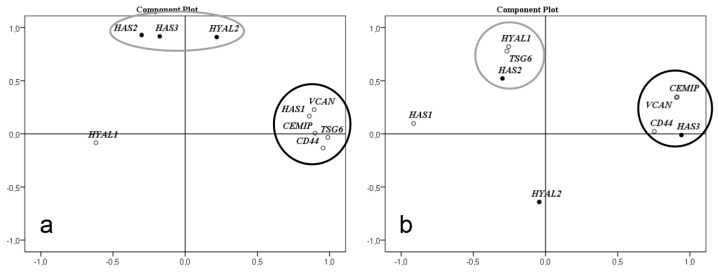
Factor analysis for correlation of gene expression levels. *HAS1-3*, *HYAL1-2*, *CEMIP*, *CD44*, *VCAN*, and *TSG6* in non-failing left wall and septum (n = 9) (**a**) and HCM myectomies (n = 5) (**b**). (**a**) In left wall and septum there are two clusters where *HAS1*, *CEMIP*, *CD44*, *VCAN*, and *TSG6* form one cluster, and *HAS2*, *HAS3,* and *HYAL2* form another. (**b**) In basal septal myectomies from HCM patients the expression levels of *CEMIP*, *CD44,* and *VCAN* formed a new correlation cluster with *HAS3*. *HAS2*, *HYAL1*, and *TSG6* formed another cluster. The levels of *HAS1* and *HYAL2* no longer correlated with any of the other genes investigated. Factor analysis was performed with the principal components method to analyze the correlation matrix and two factors were extracted.

Factor analysis of gene expression level correlation in left ventricle of non-failing hearts showed that *HYAL2*, *HAS2,* and *HAS3* formed a correlation cluster while the genes *CEMIP*, *CD44*, *HAS1*, *TSG6,* and *VCAN* formed another, as shown in Figure 3a. In HCM, the expression levels of *CEMIP*, *CD44,* and *VCAN* formed a new correlation cluster with *HAS3*. *TSG6*, *HAS2,* and *HYAL1* formed another cluster, as shown in Figure 3b. The levels of *HAS1* and *HYAL2* no longer correlated with any of the other genes investigated.

## 4. Discussion

During development of HCM, the heart is subjected to hemodynamic or metabolic stress and returns to the fetal gene program and to fetal metabolism where cells prefer usage of glucose over fatty acid as a source of energy [4,25]. Our study material is based on basal septal myectomies from HCM patients subjected to surgical intervention due to a symptomatic and advanced disease where fibrosis and disarranged morphology is already established. 

NMR analysis of metabolites in cardiac tissue from the HCM patients compared to healthy heart tissues from non-failing controls showed that fatty acids were decreased, and lactate was increased in the heart of HCM patients, indicating altered and less efficient glucose metabolism. UDP-sugar precursors, e.g., UDP-GlcNAc and UDP-GlcUA, the two substrates for HA synthesis, were also increased in the heart of HCM patients, which could be explained by the observed increase of metabolites in the HBP resulting in subsequent increase of UDP-GlcNAc. In addition, *myo*-Inositol and UDP-glucose, which are precursors for UDP-GlcUA, were also increased.

It has previously been shown that elevated levels of HA substrates enhance the synthesis of HA [8] and our metabolic results could be confirmed with an increase of low MM HA in myectomies from HCM patients compared to non-failing hearts, as shown in Figure 2f. An unexpected finding was a small but significant decrease of water content in cardiac tissue from HCM patients. Tissue accumulation of HA normally causes increased water content. Clinically human HCM is not associated with edema and all patients had end stage HCM. Possibly the low MM HA in the hypertrophic heart does not retain water in the tissue.

The factor analysis of expression level of genes involved in synthesis, degradation, and binding of HA clearly demonstrated that expression of genes involved in HA metabolism are altered in HCM. Interestingly, the expression levels of the three genes *HYAL2*, *HAS2* and *HAS3* correlated closely in the control left ventricle. Furthermore, levels of *HYAL2* and *HAS3* also correlated to amounts of low MM HA in the controls. In cardiac tissue from the HCM patients, these three genes showed significant changes in expression levels, both up and down regulated, but they had lost their mutual correlation and also to low MM HA. These clusters of gene expression levels might indicate genes involved in the same cellular process, governed by the same set of transcription factors. The pathological process of HCM induces another set of transcription factors causing different clusters of gene expression levels. This implies a common transcriptional regulation in the healthy heart which is disrupted during the development of HCM affecting the metabolism of HA, both synthesis and degradation. However, we cannot evaluate the functional significance of these gene expression clusters based on our results.

Downregulation of *HYAL2* suggests that less HA is degraded and eliminated intracellularly. However, oxidative stress and excess production of ROS is a feature of HCM, which also has been implied in degrading HA into low MM HA. Glutathione has a protective function in the attenuation of ROS in HCM. In our metabolomic analysis glutamine and glutamate, substrates for glutathione, as well as glutathione itself were increased in cardiac tissue from the HCM patients, indicating a cellular response of high content of ROS. Thus, ROS might be a possible explanation for the fragmentation and accumulation of low MM HA in the hypertrophic heart.

Based on our results, a hypothetical explanation of the increased levels of low MM HA seen in the cardiac tissue from HCM patients might be increased availability of HA substrates from increased glucose metabolism, upregulated expression of HAS3, downregulation of internal degradation by HYAL2 with subsequent extracellular fragmentation by ROS. 

We observed that the size of cardiomyocytes corresponded with the amount of low MM HA in human cardiac tissue, both in non-failing heart tissue as well as in basal septal myectomies from HCM patients, as shown in Figure 2a–e. We experimentally confirmed accumulation of low MM HA in cardiac tissue from a model of induced cardiac hypertrophy in rat, showing a rapid shift from high MM HA to low MM HA after surgery, as shown in Figure 2g,f. This suggests that fragmentation of HA into low MM HA occurs both in different parts of non-failing hearts and during the development of cardiac hypertrophy with levels corresponding to cardiomyocyte size. 

An interesting parallel is found in experimental myocardial infarction research [26]. When HA-based hydrogels with HA of different mass were injected in the infarcted area, the gel with the smallest mass HA (50 kDa) showed the most significant regeneration of myocardium and functional recovery. This further supports the hypothesis that low MM HA is not pathogenic in itself but possibly a part of a compensatory process initiated by the need of increased cardiac capacity.

An important feature in HCM is the development of arrhythmias including lethal arrhythmias. Increased fibrosis is known to disrupt the electrical conductivity between cardiomyocytes and act as a substrate for re-entrant arrhythmias [27]. In our study, we observed an increased staining intensity of HA surrounding individual cardiomyocytes in cardiac tissues from HCM patients compared to healthy controls, as shown in Figure 1c,f. Furthermore, it has been shown that high MM HA depolarizes the membrane potential in human fibroblasts, human embryonic kidney (HEK), and neurons in a concentration dependent manner which could be reversed by digestion of HA by hyaluronidase [13]. Our observations and others results [13] could indicate that a local increase of high MM HA in HCM may play a role in membrane polarizing of individual cardiomyocyte cell membranes. 

The morphology of hypertrophic myocardium is characterized by regions of seemingly normal tissue, neighboring large disarrayed cardiomyocytes with a remodeled and expanded ECM. We identified regions within expanded ECM areas with stronger HA and weaker collagen staining and vice versa. Accumulation of HA has been shown to precede the development of fibrosis, and low MM HA has been predicted to have an essential role in promoting fibrosis [12]. Low MM HA binds to the Toll-like receptor 2 (TLR2) and it has been shown that inhibition of TLR2 reduces cardiac fibrosis [28,29]. In addition, HA occurs in the myocardial infarction border zone [30] and degradation of HA with hyaluronidases in early treatment of myocardial infarction has been shown to reduce fibrosis and infarct size [31]. Possibly, areas with either more HA or collagen respectively mirrors different stages of progress of the disease and formation of fibrosis. 

Both HA’s effect on cellular action potential and on the development of fibrosis suggests involvement of HA in development of arrhythmia. In addition, the increased amount of HA contributes to the expanded ECM, thus separating cardiomyocytes within myofibrils and disrupting their cell-to-cell connection and impulse conduction, which also could be a potential risk for arrhythmia.

Cardiac energy metabolism in HCM affects the heart in several ways on a molecular level, e.g., O-GlcNAcylation and mTOR activation. Here we have introduced changes in HA metabolism as another consequence of the dysregulated glucose metabolism.

The study is limited by the availability of human cardiac tissue. Cardiac tissue from five patients and five healthy control hearts are few in a statistical view but valuable material to support results from experimental models.

In conclusion, we have, in various steps, shown that both HA molecular mass and amount changes in the development of HCM. The return to fetal energy metabolism in the HCM heart causes an increased generation of substrates for HA, which together with an altered gene expression changes the metabolism of HA. HA might add to the risk of arrhythmias in HCM and the process of regulating cardiomyocyte size seems to involve fragmentation of HA into low MM HA. The connection of glucose metabolism to HCM and the heart needs further investigation since diet and disease, e.g., diabetes mellitus, can affect the cellular uptake of glucose. This is a novel addition to the underlying mechanisms of hypertrophic cardiomyopathy.

## Figures and Tables

**Table 1:** Clinical characteristics of the patients with hypertrophic obstructive cardiomyopathy (HOCM).

Characteristics	HOCM Patients (n = 5)
Age (year)	55.4 ± 12.5
Gender (male/female)	2/3
NYHA class (II/III/IV)	1/4/0
IVSd (mm)	19.3 ± 3.8
LVPWd (mm)	12.3 ± 1.9
LVIDd (mm)	43.7 ± 3.5
LVOT (mmHg)	47.4 ± 29.8
**Medication**	
Calcium antagonist	2
Acetylsalicylic acid	4
β-antagonist	3
Aldosterone antagonist	1
Statins	3

Data are presented as the mean ± SD or number of subjects. NYHA, New York Heart Association Functional Classification; IVSd, interventricular septum in end diastole; LVPWd, left ventricular posterior wall in end diastole; LVIDd, left ventricular internal diameter in end diastole; LVOT, left ventricular outflow tract.

**Table 2:** NMR analysis of cardiac tissue from patients with hypertrophic cardiomyopathy (HCM) (n = 5) compared to healthy controls’ left wall and septum (n = 10). The variable importance in projection (VIP) score and fold change of metabolites with significant difference between HCM and non-failing left chamber wall.

	VIP Score	pos/neg Correlation	Fold Change	*P*-Value
**Sugars and related metabolites**				
UDP-glucose, UDP-GlcUA, UDP-galactose, UDP-GlcNAc	1.03	0.13	1.86	0.054 *
glucose	1.44	−0.18	0.63	0.007
**Amino acids**				
glutamate	1.83	0.22	3.04	0.002
glutamine	1.43	0.18	2.14	0.013
**Other metabolites**				
taurine	1.58	0.21	2.52	0.009
*myo*-Inositol	1.55	0.18	1.63	0.005
lactate	1.31	0.17	3.74	0.054 *
acetate	1.34	0.17	3.39	0.023
glutathion	1.53	0.19	1.77	0.007
**Fatty acids**				
CH_2_CH_2_CH_2_– lipid	1.27	−0.16	0.54	0.031
CH_2_CO– lipid	1.36	−0.17	0.55	0.017
CH_2_CH_2_CH_2_CO– lipid	1.38	−0.17	0.55	0.023
CH_2_CH_2_CH=CH– lipid	1.39	−0.17	0.64	0.017
CH_3_CH_2_– lipid	1.43	−0.18	0.58	0.017

Variable importance in projection (VIP). (*) *P* values > 0.05. Fold change was calculated by using the means in the included tissues and compared with a non-parametric Mann–Whitney test. For metabolomics statistics, non-parametric Mann–Whitney U test with Benjamini–Hochberg correction using in-house software written and compiled in MATLAB (MathWorks Inc., MA, USA) was used.

**Table 3:** Gene characteristics and results of gene expression analysis. Changes in relative amount of low and high MM HA, cardiomyocyte (CM) area, and content of water in basal septum myectomies from HCM patients (n = 5) compared to non-failing septum and left ventricle (LV) (n = 10).

Gene	Description	Applied Biosystems	Fold Change in Myectomized vs. Healthy LV	*P*-Value
		Assay Number		
*HYAL2*	Hyaluronidase 2	Hs01117343_g1	↓ 1.8	0.003
*HAS2*	Hyaluronan synthase 2	Hs00193435_m1	↓ 3.1	0.030
*HAS3*	Hyaluronan synthase 3	Hs00193436_m1	↑ 2.1	0.002
**Change in:**				
Low MM HA	Relative change in amount of low MM HA		↑ 3.1	0.045
High MM HA	Relative change in amount of high MM HA		↑ 1.6	0.171
CM area	Relative change in CM area		↑ 1.7	0.000
Water content in cardiac tissue	Relative change in water content/mg tissue		↓ 1.3	0.048

Statistical analysis using non-parametric Mann–Whitney U test. Arrows indicate up or down regulated.
